# Supplementary material for: Unravelling the Inflammatory Processes in the Early Stages of Diabetic Nephropathy and the Potential Effect of (Ss)-DS-ONJ
Source: Int J Mol Sci. 2022 Jul 30;23(15):8450. doi: 10.3390/ijms23158450 (PMC9368839; doi:10.3390/ijms23158450)
Supplement: Supplementary file 1 [file ijms-23-08450-s001.zip › ijms-1832760-supplementary.pdf]

Table S1. Antibodies used for western-blot and immunofluorescence assays

ANTIBODY	COMPANY	REFERENCE	DILUTION OF USE
**iNOS**	Abcam (Cambridge, UK)	ab15323	1:1000
**Caspase-1 (p-10)**	Abcam (Cambridge, UK)	ab 179515	1:1000
**E-cadherin**	BD Bioscience (Madrid, Spain)	BD610182	1:500 (WB)/ 1:250 (IHC)
**LC3-I/II**	Cell Signaling Technologies (Danvers, MA, USA)	12741	1:1000
**Pp38-MAPK**	Cell Signaling Technologies (Danvers, MA, USA)	9211	1:1000
**p38-MAPK**	Cell Signaling Technologies (Danvers, MA, USA)	9212	1:1000
**P SAPK/JNK**	Cell Signaling Technologies (Danvers, MA, USA)	9255	1:1000
**SAPK/JNK**	Cell Signaling Technologies (Danvers, MA, USA)	9252	1:1000
**ZEB-1**	Cell Signaling Technologies (Danvers, MA, USA)	70512	1:1000
**Snai-1**	Cell Signaling Technologies (Danvers, MA, USA)	3879	1:1000
**Slug**	Cell Signaling Technologies (Danvers, MA, USA)	9585	1:1000
**p62**	MBL (Japan)	PM045	1:1000
**α-SMA**	Sigma-Aldrich (St Louis, MO, USA)	A2547	1:500 (WB)/ 1:500 (IHC)
**p65**	Santa Cruz Biotechnology (Dallas, Texas, USA)	sc-372	1:500
**α-tubulin**	Sigma-Aldrich (St Louis, MO, USA)	T-5168	1:15000
**Anti-rabbit IgG-HRP**	Sigma-Aldrich (St Louis, MO, USA)	A0545	1:5000
**Anti-mouse IgG-HRP**	Sigma-Aldrich (St Louis, MO, USA)	A9044	1:5000
**anti-rabbit Alexa 488**	Invitrogen (Carlsbad, CA, USA)	A32731	1:500

Table S2. Rat and mouse probes used for RT-qPCR

Taq-Man GENE EXPRESSION ASSAYS	COMPANY	REFERENCE
**Rat:**<br>***Nos2***<br>***Tnfa***<br>***Il1b***<br>***Nlrp3***<br>***Il18***<br>***Cadherin-1***<br>***Acta-2***	Applied Biosystems, Spain	 Rn00561646_m1<br>Rn99999017_m1<br>Rn00580432_m1<br>Rn04244620_m1<br>Rn01422083_m1<br>Rn00580109_m1<br>Rn01759928_g1

Tgfb<br>***actinb***		Rn00572010_m1<br>Mm00607939_s1
**Mouse:**<br>***Nos2***<br>***Tnfα***<br>***Il1β***<br>***Nlrp3***<br>***Il18***<br>***Cadherin-1***<br>***Acta-2***<br>***Tgfb***<br>***actinb***	Applied Biosystems, Spain	 Mm00440502_m1<br>Mm00443258_m1<br>Mm00434228_m1<br>Mm00840904_m1<br>Mm00434225_m1<br>Mm01247357_m1<br>Mm00725412_s1<br>Mm01178819_m1<br>Mm00607939_s1
